# The assessment of molecular dynamics results of three-dimensional RNA aptamer structure prediction

**DOI:** 10.1371/journal.pone.0288684

**Published:** 2023-07-27

**Authors:** Bejo Ropii, Maulidwina Bethasari, Isa Anshori, Allya Paramita Koesoema, Wervyan Shalannanda, Ardianto Satriawan, Casi Setianingsih, Mohammad Rizki Akbar, Reza Aditama

**Affiliations:** 1 School of Electrical Engineering and Informatics, Bandung Institute of Technology, Bandung, West Java, Indonesia; 2 Department of Pharmacy, Universitas Muhammadiyah Bandung, Bandung, West Java, Indonesia; 3 Research Center for Nanosciences and Nanotechnology (RCNN), Bandung Institute of Technology, Bandung, West Java, Indonesia; 4 Department of Computer Engineering, School of Electrical Engineering, Telkom University, Bandung Regency, West Java, Indonesia; 5 Department of Cardiology and Vascular Medicine, Faculty of Medicine, Universitas Padjadjaran and Dr. Hasan Sadikin General Hospital, Bandung, West Java, Indonesia; 6 Biochemistry Research Group, Faculty of Mathematics and Natural Sciences, Bandung Institute of Technology, Bandung, West Java, Indonesia; Wake Forest University, UNITED STATES

## Abstract

Aptamers are single-stranded DNA or RNA that bind to specific targets such as proteins, thus having similar characteristics to antibodies. It can be synthesized at a lower cost, with no batch-to-batch variations, and is easier to modify chemically than antibodies, thus potentially being used as therapeutic and biosensing agents. The current method for RNA aptamer identification in vitro uses the SELEX method, which is considered inefficient due to its complex process. Computational models of aptamers have been used to predict and study the molecular interaction of modified aptamers to improve affinity. In this study, we generated three-dimensional models of five RNA aptamers from their sequence using mFold, RNAComposer web server, and molecular dynamics simulation. The model structures were then evaluated and compared with the experimentally determined structures. This study showed that the combination of mFold, RNAComposer, and molecular dynamics simulation could generate 14-16, 28, or 29 nucleotides length of 3D RNA aptamer with similar geometry and topology to the experimentally determined structures. The non-canonical basepair structure of the aptamer loop was formed through the MD simulation, which also improved the three-dimensional RNA aptamers model. Clustering analysis was recommended to choose the more representative model.

## Introduction

Aptamers are single-stranded DNA or RNA that bind to specific targets such as proteins or other biomolecules with high affinity. The 3D architectures of aptamers influence its binding affinity similar to the characteristics of monoclonal antibodies [1–3]. Compared to monoclonal antibodies, aptamers are less immunogenic, easier to mass-produce at lower costs, and have lower batch variation [4]. In addition, aptamers have a wider spectrum of target ligands than monoclonal antibodies, including ions and organic dyes. Therefore, aptamers have wide applications as biosensors as well as therapeutic agents. Another advantage of aptamers is that the time required for development is around 2–8 weeks, much shorter than antibodies which take more than 6 months [4].

In 1990, a selection process for aptamers named Systematic Evolution of Ligands by Exponential Enrichment (SELEX) was developed, which remains the gold standard for aptamer selection [5, 6]. In this method, the selection of aptamers is carried out in vitro from a collection of aptamers that can reach 10^14^-10^15^ variants. The method requires many cycles or is highly iterative because it involves several cycles of the oligonucleotide exposure process to the target and modification of the aptamer in vitro. Therefore, it is considered inefficient because it requires more time and effort. A great deal of research has been carried out to develop innovative techniques to produce aptamers with consistently high performance, efficiency, resource savings, and a greater chance of success [7, 8]. One of the efforts to improve the efficiency of the aptamer selection and analysis process steps is through bioinformatics. In principle, the method performs a selection process using a virtual oligonucleotide candidate library (10^14^—10^15^ variants). Each candidate will be selected based on the predicted affinity for the target obtained in-silico. The method has been developed by Yoruslav et al. [2, 9]. Several three-dimensional structures of RNA prediction platforms have been developed, such as RNAComposer, JAR3D, and iFoldRNA; all three were web-based servers while the other platforms which need local installation include Rosetta, RMdetect, and RAGTOP [10]. Among those platforms, RNAComposer offers several advantages for generating 3D RNA structures, including a user-friendly interface, high accuracy with an average RMSD of 1.7 A, fast computation, ability to handle large RNAs, ability to incorporate experimental data, and availability as a web-based application for easier access.

In this study, we used RNAComposer, a web server to predict the experimentally determined structures of RNA aptamer from the Protein Data Bank (PDB) database. The framework includes two-dimensional structure prediction using mFold, 3D structure generation using RNAComposer, and structure refinement using Molecular Dynamics (MD) simulation. We also utilized public software structure evaluation, analysis, and visualization, including RNApdbee and UCSF Chimera. The MD simulation has successfully improved the structural prediction of RNA aptamers.

## Methods

### RNA aptamer search and three-dimensional structure prediction

The experimentally determined three-dimensional structure of RNA aptamers was searched in the PDB database using “Aptamer” as the keyword. In this study, we focused specifically on single RNA aptamers. The exclusion criteria were structures with ligands, protein, modified nucleotides, quadruplex, triplex, duplex, or belonging to the DNA aptamer class. QGRS Mapper was used to predict the presence of quadruplex forming G-rich in aptamer sequences. The RNA aptamers which fulfill the inclusion and exclusion criteria were used, and the models of the 3D structure were generated. mFold web server was used to predict the secondary structure of each RNA aptamer. The dot-bracket results from mFold and its sequence were used as the input for the 3D structure generation using RNAComposer.

### Molecular dynamics simulation

MD simulation was performed using GROMACS [11] and CHARMM36 force field [12]. Each system consisted of an aptamer in TIP3P as a water model and was neutralized by adding the appropriate number of ions (sodium or chloride). Periodic boundary conditions (PBCs) were applied to the system in all the spatial directions. LINCS algorithms were used, and all hydrogen bonds were constrained. A 1.2 nm distance cutoff was used for the short-distance electrostatic and van der Waals interaction. Particle Mesh Ewald algorithm (PME) was used to calculate the long-range electrostatic forces. The steepest descent algorithm was used to minimize the system’s energy. The system was then allowed to reach an equilibrium state through the NVT ensemble using the V-Rescale thermostat at 300K, then through the NPT ensemble using the Parrinello-Rahman barostat at 1 atm. The simulation was performed for 100 ns.

## Results and discussion

### Three-dimensional structure prediction

**Fig 1:**
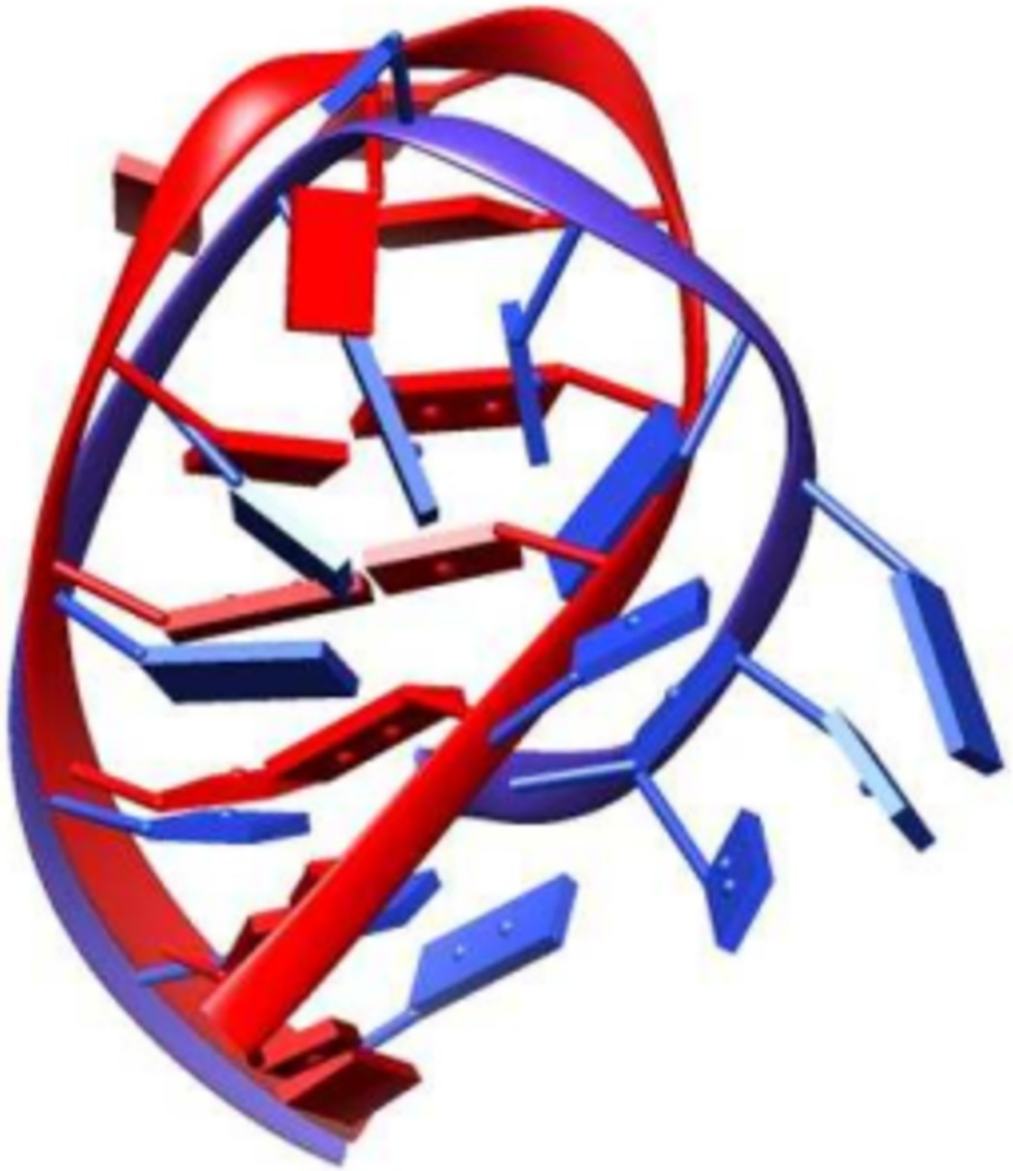
Alignment of 3D predicted structure (colored blue) and the corresponding experimentally solved structures from PDB database (colored red) for the 5 ssRNA aptamers used in this study. Each structure was labeled by its PDB ID, nucleotides (nt) number, and the calculated RMSD values (in Angstrom).

Our search of RNA aptamers using “Aptamer” as the keyword in the PDB database resulted in 351 structures, of which five aptamers matched our inclusion and exclusion criteria. The aptamers are listed in Table 1, and each aptamer has a different target protein. The length of aptamers was 14, 15, 16, 28, or 29 nucleotides. Each aptamer has one or more non-canonical base pairs at the end of the internal loop. All structures were solved by NMR spectroscopy. The aptamers were derived or obtained from modified structures of in vitro selection (SELEX) except for 2EVY, which is a fragment of poliovirus 59-clover-leaf ssRNA [3, 13–16]. The aptamer sequences in Table 1 were used to construct the 3D model by using mFold web server and RNAComposer. To evaluate the predictive success of the 3D RNA aptamer models, the constructed models must be geometrically and topologically as close as possible to the experimentally determined structure. It was assumed that the crystal structure or NMR structure is correct within the limitations of the experimental methods. The accuracy of the proposed model was assessed by aligning the predicted structure with the corresponding ssRNA aptamer structure downloaded from the PDB database. The degree of similarity was measured based on the calculated Root Mean Square Deviation (RMSD) of the whole aptamer between each pair of structures [17]. Fig 1 shows the overlay of each predicted 3D structure using mFold and RNAComposer with the corresponding NMR structures from the PDB database, including the RMSDs values. RNAComposer utilized the machine translation principle and operated on the RNA FRABASE, an engine with a database to search the three-dimensional fragments within 3D RNA structures using the sequence and the dot-bracket notation as input [18, 19]. The RMSD calculation, visualization, and hydrogen bond analysis were evaluated using UCSF Chimera. The RMSDs values of the predicted structures compared to the corresponding reference range from 2.19 Å (PDB ID: 1XWP) for the shortest sequences to 12.942 Å (PDB ID: 2LUN), with an average value of 6.292 Å. Overall 3D aptamer models were similar to their corresponding reference structure, except for 2LUN, which had an RMSD value of 12.94 Å. Furthermore, 3D structures predicted by RNA Composer were named (PDB-ID)-RC (for example, 1XWP-RC), while structures derived from RCSB were named (PDB-ID)-Ref (for instance, 1XWP-Ref). Atomistic MD simulations were conducted to improve and refine the structural predictions for the five aptamers.

**Table 1:** The results for the selection of ssRNA candidates from Protein Data Bank database.

No.	PDB ID	Length (base)	Sequence	Target	Non-Canonical Base Pair
1	1XWP	15	GGAGAUCGCACUCCA	Mammalian Translation Initiation-eIF4A	A5:A10 (end loop)
2	1XWU	16	CGAAACAUAGAUUCGA	Mammalian Translation Initiation-eIF4A	A5:A11 (end loop)
3	2EVY	14	GGUAUGCUAGUACC	The Viral Protease: 3CD	G6:A9 (end loop)
					U5:G10 (end loop)
4	2LUN	28	GGGCAGUGAUGCUUCGGCAUAUCAGCCC	Bacterial Ribosomal Protein S8	U7:U22 (internal loop)
					G8:A21 (internal loop)
5	2JWV	29	GAUACUUGAAACUGUAAGGUUGGCGUAUC	NF-iB Transcription Factor	A9:G22 (internal loop)
					G14:A17 (end loop)
					U13:G18 (internal loop)

### Three-dimensional structure refinement using MD simulations

**Fig 2:**
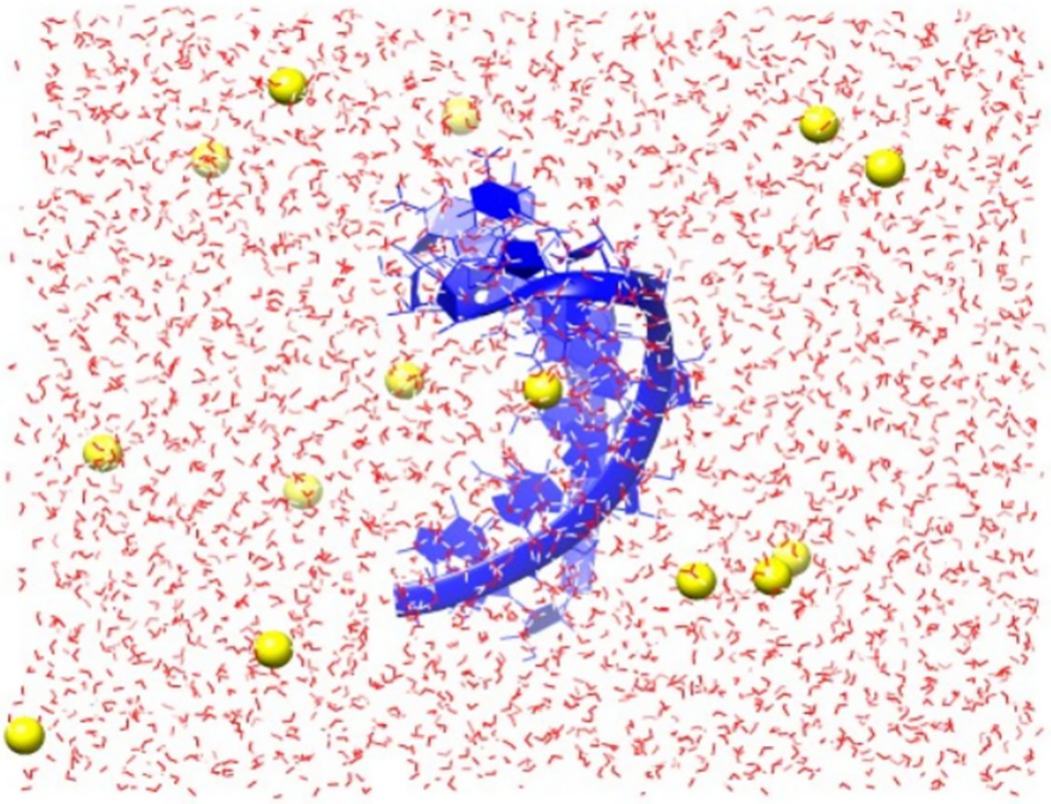
Starting configuration of the MD simulation of the 1XWP model. The aptamer molecule (colored blue) was solvated in water (colored red) and the system was neutralized with 14 sodium ions (colored yellow).

We have performed MD simulations using GROMACS to improve the structural predictions for the 5 aptamers and study the dynamics of the systems. We conducted individual MD simulations of 5 aptamer models or 5 aptamers from reference structures in a water solution model neutralized by Sodium ions. As a representative, the starting configuration of 1XWP simulation with 3142 water molecules and 14 Sodium ions was illustrated in Fig 2.

**Fig 3:**
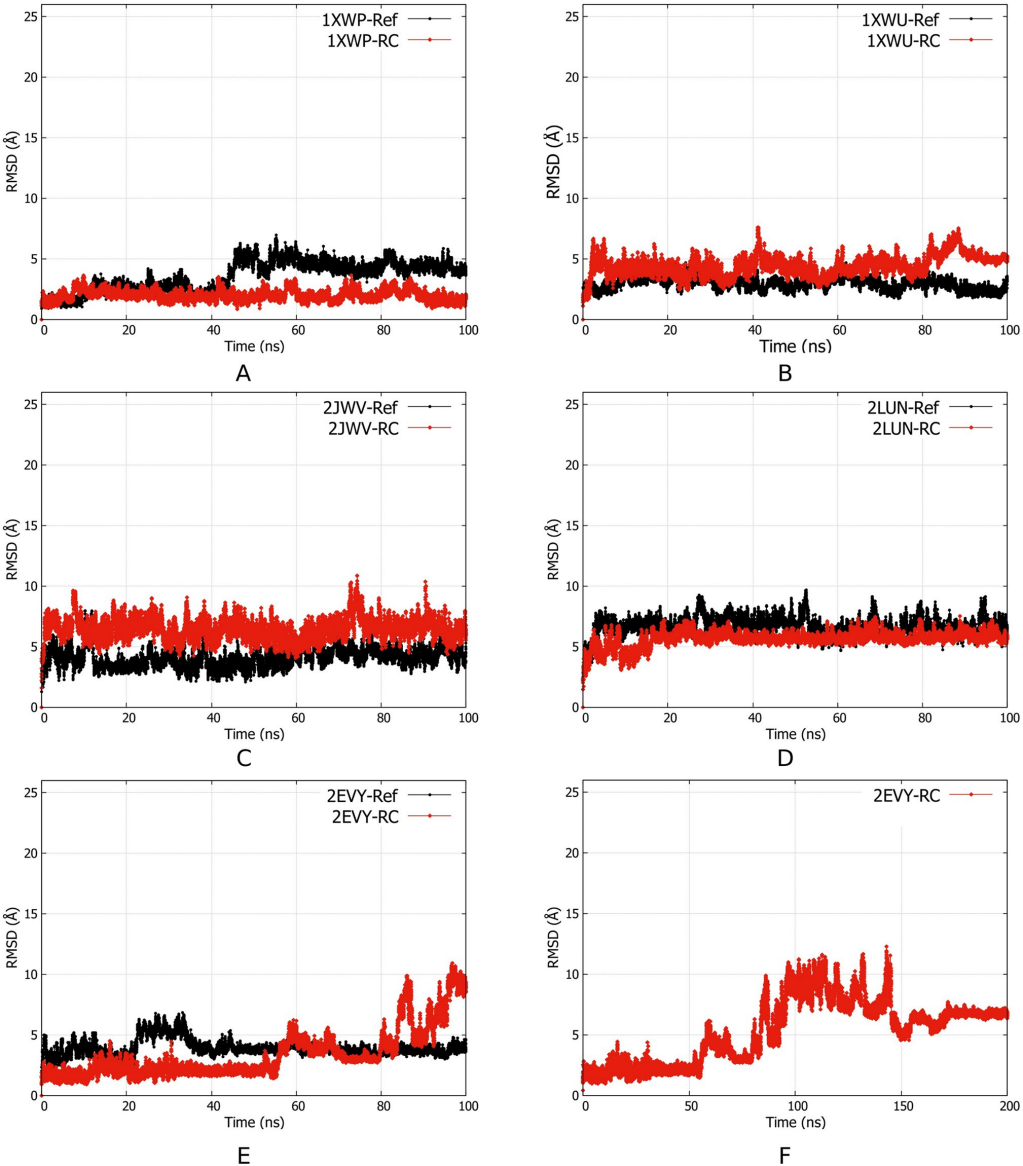
(A-E) The evolution of RMSDs towards time for the aptamer structures with respect to the first structure (time 0). (F) RMSDs evolution of 2EVY-RC after the simulation was extended to 200 ns. It can be seen that the RMSD becomes stagnant after 170 ns.

**Fig 4:**
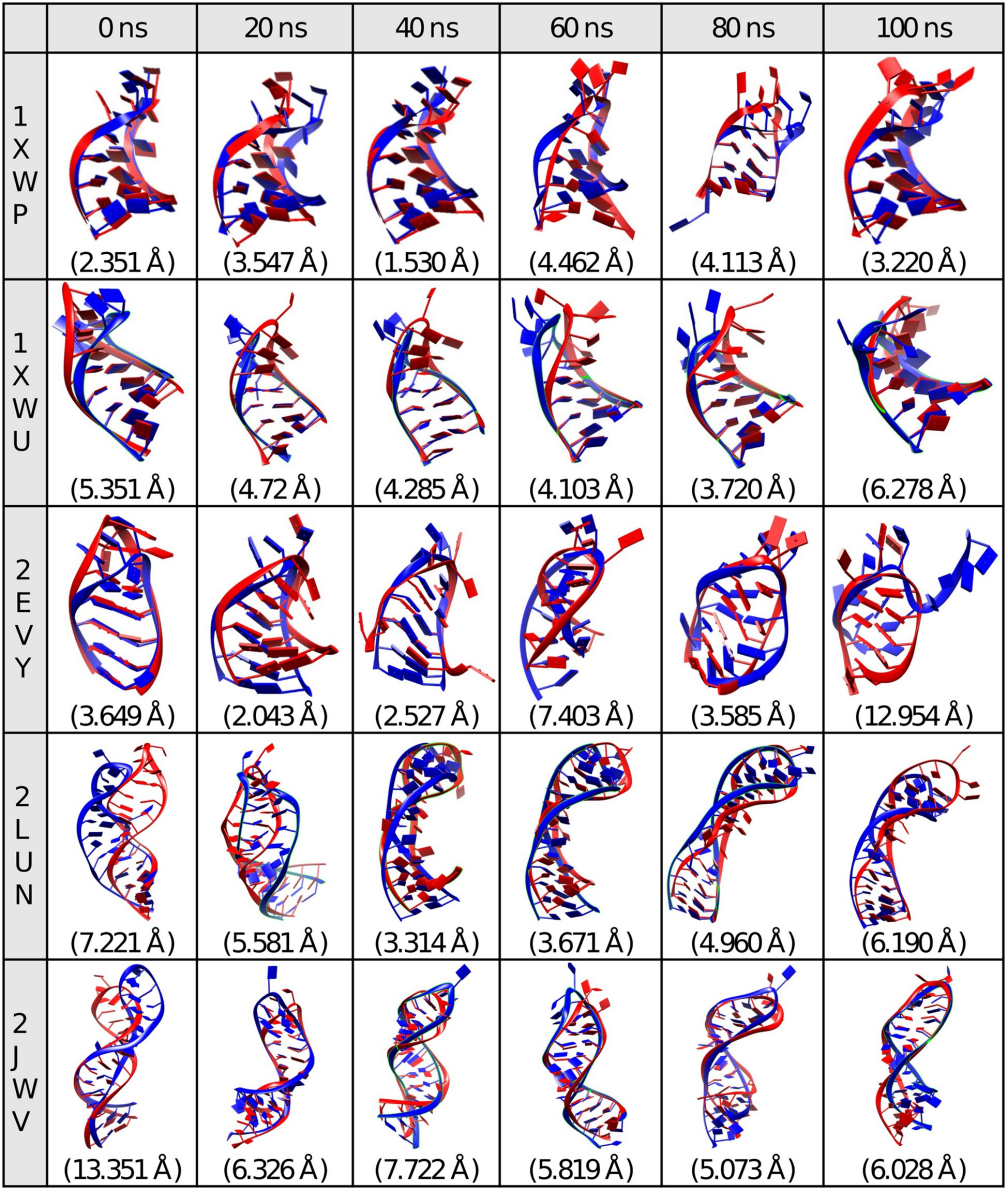
The overlays of 3D predicted structure (colored blue) and the corresponding experimentally solved structures from the PDB database (colored red) for the five aptamers at 0, 20, 40, 60, 80, and 100 ns during the MD simulation.

The RMSD of the sugar-phosphate backbone was calculated to explore the deviation of each structure with respect to its first structure (Fig 3). RMSD provided information on the conformational flexibility and structural deviation of each RNA aptamer model or reference structure. Fig 3 showed that both predicted and reference structures have similar time-dependent behavior except for 2EVY. During 100ns MD simulation, all aptamer models had similar RMSD values to the corresponding reference structures, except for the 2EVY, which started to fluctuate at 80 ns. Therefore, we extended the 2EVY simulation to 200ns for further investigation (Fig 3F). It was observed that 2EVY was stable for the last 50ns during the 200ns MD simulation. A snapshot was taken at 0 ns, 20 ns, 40 ns, 60 ns, 80 ns, and 100 ns to visually analyze the conformation change of the aptamer model compared to the reference structure during the MD simulation. Fig 4 showed that all aptamer models evolved during the 100ns length of MD simulation, particularly 2EVY, which was characterized by the higher RMSD value and hairpin loop opening at 100ns.

Visual inspection of the 2EVY structure in Fig 4 showed that the RMSD fluctuation observed in 2EVY at 80–150 ns (Fig 3F) was due to the opening of the hairpin loop structure. By the end of the 200ns simulation, the 2EVY structure became more similar corresponding to the reference (Fig A1 in S1 Appendix) with RMSD 6.801 Å. In Fig 3, the RMSD of 2EVY-RC began to fluctuate towards the end of the simulation (100 ns), indicating that a longer simulation was required to stabilize the structure. The other aptamers have shown a stable structure as shown by its RMSD value.

Fig 4. The overlays of the 3D predicted structure (colored blue) and the corresponding experimentally solved structures from the PDB database (colored red) for the five aptamers at 0, 20, 40, 60, 80, and 100 ns during the MD simulation.

To investigate the structure difference between aptamer models during the 100 ns simulation with the crystal structure, we calculated the RMSD values of each aptamer model with respect to the crystal structure of the reference structure at several time points (Table 2). Based on Table 1, 1XWP became more similar with its crystal structure after 100ns MD simulation, while 1XWU, 2LUN, and 2JWV have a deviation under 2Å at each time point. The deviation of 2EVY after 100 ns became higher due to the opening of the hairpin loop. We extracted the representative structure of all aptamers during 100 ns MD simulation to perform further investigation.

**Table 2:** The summary of RMSD values at different time points throughout the MD simulation of the aptamer models corresponding to its crystal structures from the PDB database.

Time (ns)	RMSD (Å)				
	1XWP	1XWU	2EVY	2LUN	2JWV
0	2.11	4.82	3.47	7.96	4.37
10	3.67	3.77	3.94	9.33	5.20
20	2.63	3.72	3.76	6.92	5.25
30	2.45	4.03	3.41	8.83	5.48
40	2.82	4.20	3.44	7.14	4.01
50	2.17	4.19	3.58	7.27	4.75
60	3.00	4.50	6.04	7.61	3.59
70	2.25	4.21	5.07	7.17	4.80
80	3.12	4.62	4.86	8.27	4.54
90	1.92	5.97	5.79	8.06	4.78
100	1.47	5.30	10.05	7.10	5.03

**Fig 5:**
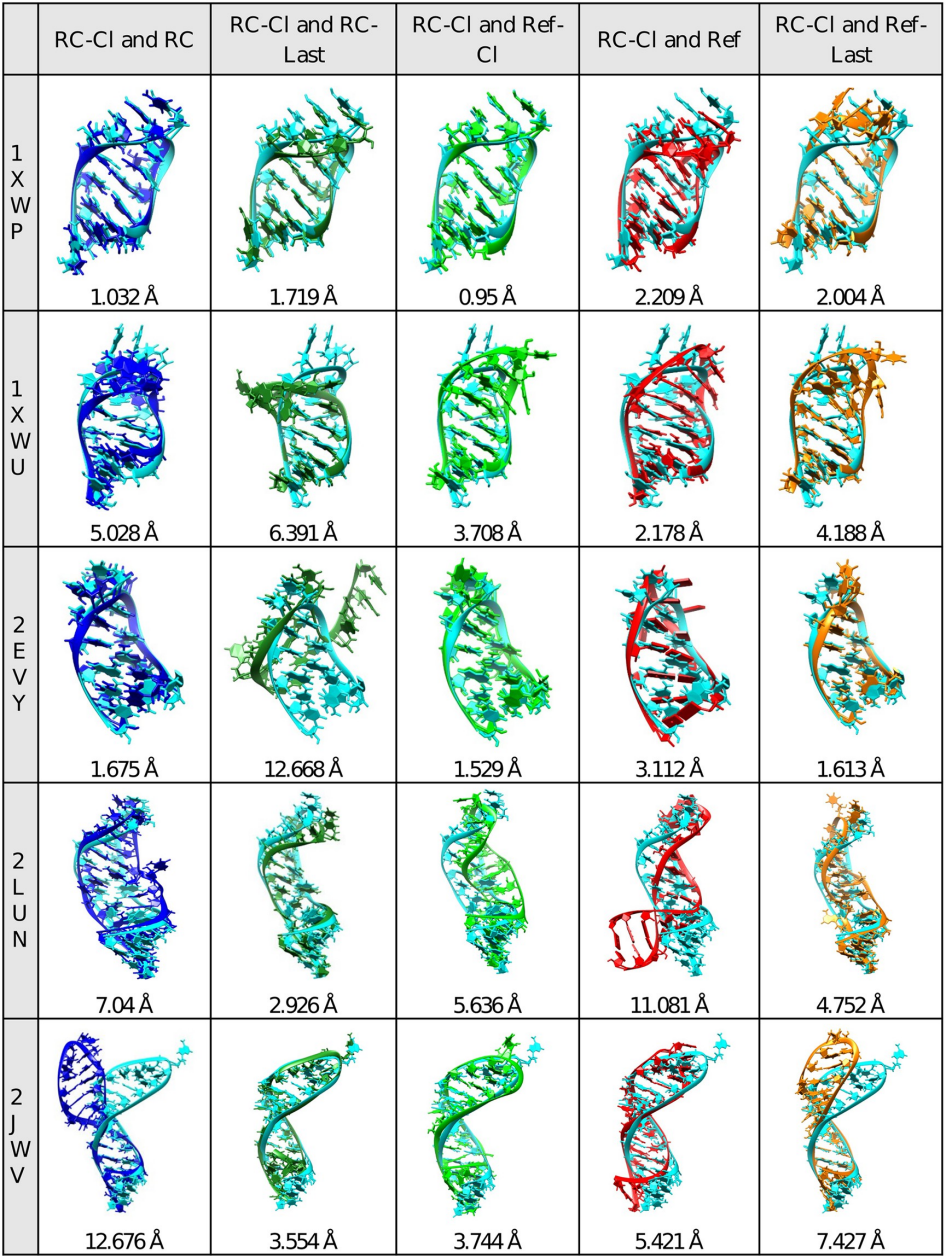
Comparison of the representative structure of aptamer model (RC-Cl, light blue) with their corresponding structure before simulation (RC, blue), after 100ns simulation (RC-last, dark green), representative of reference structure (Ref-Cl, light green), crystal structure (Ref, red), and structure of reference structure after 100ns simulation (Ref-last, orange). The RMSD of each alignment was written at the bottom of the structure (in Angstrom).

GROMACS can calculate the representative structure during the MD simulation period. All resulting frames from the 100 ns MD simulation were clustered. The cluster from simulations of both the aptamer model and reference that contain the higher cluster members were analyzed and compared. Each 100 ns simulation containing 10001 structures was used to calculate the representative structure. The RMSD between these clusters was calculated using UCSF Chimera, summarized, and illustrated in Fig 5.

RMSD distribution plot (Fig A2 in S1 Appendix) showed that all aptamer models have similar RMSD distribution with the references during 100ns MD simulation except for the 2EVY. The selected cluster of each aptamer model was compared to the initial structure before simulation, the initial structure after 100ns simulation, the crystal structure, the selected cluster from reference structure, or the reference structure after 100ns simulation. We demonstrate that the simulation improves the predicted structure, and most of the conformation of the predicted and reference structure are similar during simulation. Compared to the end conformation, those from major clusters resulted in less RMSD corresponding to their reference and the major cluster of the reference. The less RMSD also applies when compared to those of RC-derived structures. Therefore, structures from major clusters can be used to represent predicted structures. From these results, we found that most of the aptamer conformation during the simulation of the predicted and the corresponding reference structure is similar.

For further analysis, we conducted a comparison of secondary structures of each conformation derived from cluster, reference structure, and RNA Composer. The secondary structure was generated by using web-based software http://rnapdbee.cs.put.poznan.pl/. RNA PDBEE derives secondary structure topology from the tertiary structure of RNA and/or from the list of base pairs. This process is based on the algorithm that iteratively unknots the RNA structure, saves partial information about knotting order, to finally merge intermediate results and encode the RNA topology [20, 21]. The obtained secondary structure of the major cluster from the simulation of reference and model were compared and summarized in Fig A3 in S1 Appendix. All five aptamers contain non-canonical base pairs which were also explained in the main publication of each corresponding structure. For 1XWU, 1XWP, and 2EVY, the non-canonical base pairs were in the loop, and all of them could be found in the aptamer model, except for A5:A11 in 1XWU. In this study, the initial structure of 2EVY-RC and 2EVY-Ref was different in A9:G6 base conformation, which resulted in different initial loop conformation Fig A4 in S1 Appendix. A9:G6 basepair in 2EVY-RC had trans sugar hoogsteen, while in 2EVY-Ref had cis sugar hoogsteen (Fig A3 in S1 Appendix). Both trans and cis sugar hoogsteen are commonly found in nucleotide structure [22–24]. At 60 ns during the 100 ns MD simulation, we observed a distinct sterical difference of A9 in the predicted and reference structure, which affected the neighbor U5:G10 wobbly base pair. The difference in the loop (base 5–10) conformation consequently affects the base pair formation in the stem (base 2–4 and 11–13). Based on the visual inspection of the simulation results, the difference in stem conformation is followed by the different persistency of syn glycosidic bond angles formation in C14 which were initially observed at 60 ns (Fig A4(B) in S1 Appendix). The syn angle in C14 continuously persisted in the 2EVY-RC simulation and did not form base pair after 70ns. Meanwhile, C14 in 2EVY-Ref formed a base pair with G1, resulting in an intact stem structure after 70 ns (Fig A4(C) in S1 Appendix). Therefore, although the non-canonical base pairs (A9:G6) were predicted in the 2EVY-RC, the sterical structure of the aptamer loop was different compared to 2EVY-Ref which then affected the overall conformational changes during the simulation (Fig A4(D) in S1 Appendix). Both non-canonical base pairs in 2LUN-RC were not predicted, while 2JWV-RC predicted 2 out of 3 non-canonical base pairs. This might cause 2JWV-RC to be more similar to the corresponding reference structures compared to 2LUN-RC.

During the simulation, both 2LUN-RC-Cl and 2LUN-Ref-Cl formed a non-canonical base pair in the end-loop resulting in the loop structure being similar to those of the reference structure. On the other hand, both 2LUN-RC-Cl and 2LUN-Ref-Cl formed a non-canonical base pair in the end-loop resulting in the loop structure being similar to those of the reference structure. Hence the RMSD value corresponding to 2LUN-Ref-Cl of 2LUN-RC-Cl is lower than those of 2LUN-RC. At the beginning of the simulation, the RMSD of 2JWV-RC and 2JWV-Ref indicate similarity, but the loops are notably different according to visual inspection. The comparison of 2JWV-RC-Cl and 2JWV-Ref-Cl showed that both internal and end loops were visually similar, which manifested to the improvement of RMSD value. Hence, it is essential to enhance the precision of loop and non-canonical base pair prediction when generating 3D structures. This is because any newly discovered non-canonical base pairs are not always observed in short molecular dynamic simulation. The inaccuracy of three-dimensional structure of the loop and non-canonical base pair could potentially result in the overlooking of instabilities throughout the simulation. The accuracy of predicting the 3D structure of RNA molecules using computational tools may be affected by various factors, such as the length of the RNA sequence and the presence of non-canonical base pairs. Longer RNA sequences typically have a greater number of nucleotides, which can increase the complexity of the structure and the likelihood of errors or inaccuracies in the prediction. Furthermore, some RNA molecules contain non-canonical base pairs, which are formed by interactions between nucleotides that are not typically paired according to the Watson-Crick base pairing rules. Non-canonical base pairs can be more difficult to predict and may not be included in standard computational tools. To improve the accuracy of predicting the 3D structure of RNA aptamers, it is suggested to incorporate non-canonical base pair prediction in the computational process. This can help to account for the presence of non-canonical base pairs and improve the accuracy of the predicted 3D structure.

## Conclusions

We have faithfully predicted the three-dimensional structure of five RNA aptamers using mFold, RNAComposer, and MD simulation which were geometrically similar to the experimentally determined structures available in the Protein Data Bank databases. The limitation of mFold and RNAComposer was the inability to predict the non-canonical base pair present in the aptamer structure. This study showed that atomic MD simulation could be used to improve the predicted aptamer model by extracting the representative structure of the trajectory. This approach worked for the five aptamers RNA aptamers which contain stem-loop and internal loop motifs with 14–16, 28, or 29 nucleotides length.

## Supporting information

S1 Appendix (PDF)
