# Supplementary material for: The assessment of molecular dynamics results of three-dimensional RNA aptamer structure prediction
Source: PLoS One. 2023 Jul 27;18(7):e0288684. doi: 10.1371/journal.pone.0288684 (PMC10373999; doi:10.1371/journal.pone.0288684)

Appendix

A1 Fig. The aligned structure of 2EVY at the end of 200 ns simulation (colored blue) with the corresponding reference structure (colored red).

Fig A1

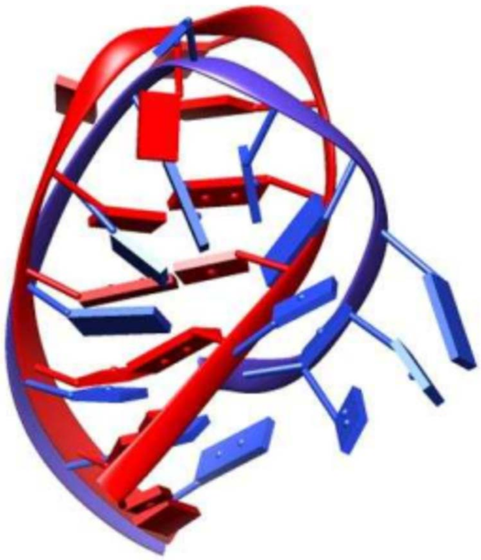

A2 Fig. RMS distribution of each structure simulation. The graph illustrates the number of structures with similar RMSD with the corresponding first structure in the reference simulation. The distribution of predicted structure is represented by the red line and those of reference structure by a black line.

Fig A2

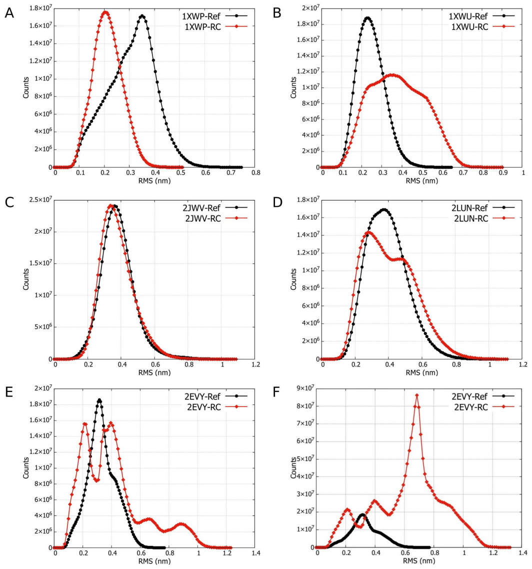

A3 Fig. **The secondary structure of major cluster from predicted structures during simulation (RC-Cl), predicted structure before simulation (RC), last structure of predicted structure simulation (RC-last), a major cluster of reference structure during simulation (Ref-Cl), reference structure from pdb (Ref), and last structure of reference structure simulation (Ref-Last).**

Fig A3

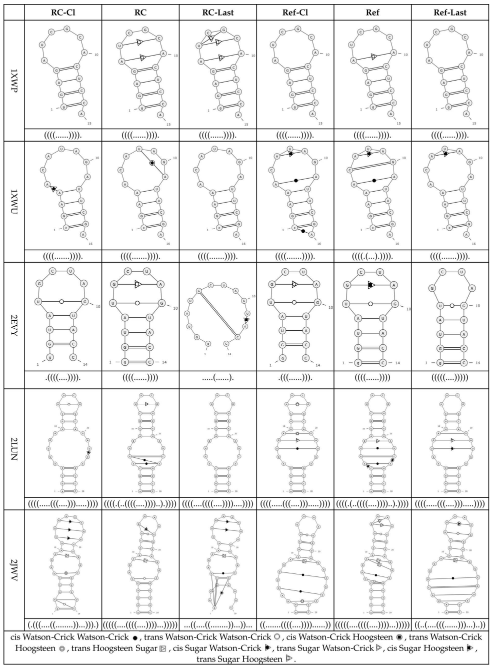

A4 Fig. The conformation of A9 (light green), G6 (Light blue), U5:G10 base pair (pink), and C14 (yellow) in the reference structure (colored) and predicted structure (blue) of 2EVY. A) The initial structure, B) Structure at 60 ns, C) Structure 70 ns.

Fig A4

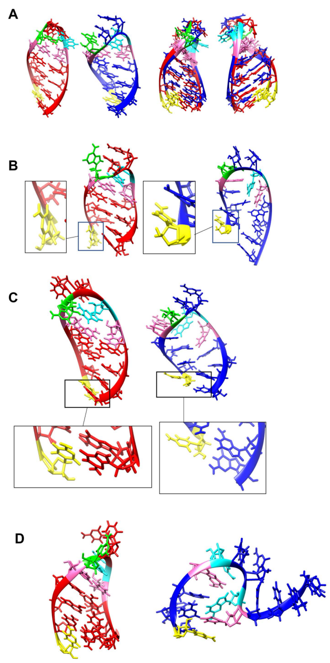

Supplement: S1 Appendix — (PDF) [file pone.0288684.s001.pdf]
